# Self-Mechanical Anal Dilatation: A Simple Trick to Minimize Postoperative Pain and Stenosis Following Hemorrhoidectomy With Radiofrequency

**DOI:** 10.3389/fsurg.2021.711958

**Published:** 2021-08-30

**Authors:** Chiara Eberspacher, Pietro Mascagni, Kenneth Paul Zeri, Lisa Fralleone, Gabriele Naldini, Domenico Mascagni

**Affiliations:** ^1^Department of Surgical Science, Policlinico Umberto I, Sapienza University of Rome, Rome, Italy; ^2^IHU-Strasbourg, Institute of Image-Guided Surgery, Strasbourg, France; ^3^Villa Tiberia Hospital, Rome, Italy; ^4^Proctological and Perineal Surgical Unit, AOU, Cisanello University Hospital, Pisa, Italy

**Keywords:** hemorrhoidectomy, postoperative pain, stenosis, dilation, constipation

## Abstract

**Aim:** Hemorrhoidectomy is still the most effective surgical treatment for hemorrhoidal disease, but it is, however, associated with complications such as pain and stenosis. We proposed to break the “vicious circle” of “pain–sphincteric spasm–stenosis–pain” with the postoperative use of self-mechanical anal dilation.

**Methods:** We retrospectively analyzed patients with hemorrhoidal disease presenting with a minimum of piles of three quadrants, treated with radiofrequency hemorrhoidectomy between January 2018 and December 2019. All the patients that at 3 weeks presented sphincteric spasms with painful defecation, were considered. Thirty-nine patients performed the cycle of self-mechanical anal dilation (Group A). This group was 1:1 matched with homogeneous patients from our historical cohort of patients (Group B). The primary endpoint was the pain evaluation, secondary endpoints: WCS, overall satisfaction of the patient, anal sphincter spasm, scarring, and the incidence of postoperative stenosis.

**Results:** In Group A mean VAS was 3.25 after 14 days of application and 1.15 at the end of the application. In Group B mean VAS was persistently higher, with a mean VAS of 5 (*p* = 0.000002) and 3.38 (*p* = 0.0000000000009). In Group A we observed an improvement of symptoms at the end, with a good overall satisfaction (Group A 7.4 vs. Group B 5.9; *p* = 0.0000007) and a better mean WCS (Group A WCS 2.8 vs. Group B WCS 4.18; *p* = 0.0001). Stenosis was observed in 3/39 patients of Group B (7.7%).

**Conclusions:** Self-mechanical anal dilation improves the pain in the late postoperative course, minimizing the risk of anal stenosis.

## Introduction

Hemorrhoidectomy is still the most effective treatment for grade hemorrhoidal disease (1). However, it is associated with a higher rate of postoperative (PO) complications when compared to other procedures, such as hemorrhoids ligation, sclerotherapy, or doppler-assisted artery ligation, usually preferred for not prolapsing diseases. Stapled hemorrhoidectomy, indicated for hemorrhoid prolapse, is also associated with less PO pain and with a lower percentage of anal stenosis, at the cost of other potentially life-threatening complications (2) or recurrence (3). One hateful complication after Milligan Morgan hemorrhoidectomy is the persistent PO pain associated with anal sphincter spasm. This often occurs when three or more piles are excised, even if a radiofrequency device is used (4). Furthermore, pain, inducing involuntary sphincteric anal spasms during defecation, can cause repetitive traumas to the mucosa with a consequent more difficult healing (5). Stenosis of the anal canal, which is another consequence of wide excision, reduces the compliance of all the anorectum, with an alteration in bowel movements, difficulty during defecation, anal bleeding, and an increase in stool frequency, affecting the quality of life of the patient for the worst.

Anal dilators are widely used for anal fissures, despite some recent works contradicting their efficacy (6). We hypothesize that anal dilators could be of use not only for the mechanical break of stenosis but also to teach the patient to relax the sphincter, avoiding the anal spasm, minimizing the daily trauma of defecation, and the associated pain. Thus, in patients suffering from persistent pain and anal spasms following excisional hemorrhoidectomy, we proposed to break the vicious circle of “pain—sphincteric spasm—stenosis—pain” with the use of an anal dilator.

The present study aimed to investigate whether the use of anal dilatators in the postoperative course, could decrease pain, improve the quality of defecation and avoid the risk of stenosis, after a 3 or 4 quadrant radiofrequency hemorrhoidectomy.

## Methods

We retrospectively analyzed all patients with IV grade hemorrhoidal disease, presenting with 3 or 4 quadrants piles treated with excisional radiofrequency hemorrhoidectomy at our specialized Proctologic Surgery Unit, between January 2018 and December 2019. All the patients that, at 3 weeks presented sphincteric spasms with painful defecation, were considered for the study. The first dilation was performed at the third PO week when the risk of severe bleeding was over. The minimum period of follow-up was 1 year.

Patients underwent hemorrhoidectomy by the same surgical team; they were operated in the lithotomy position. A simple enema was given preoperatively. The anus was dilated with an Eisenhammer retractor: all the piles were separated from the skin with an initial incision using a monopolar scalpel. When the correct surgical plan on the internal sphincter was revealed, excisional hemorrhoidectomy was completed with Ligasure™, a radiofrequency device, without any pedicles ligature, stitches, or suture. No internal sphincterotomy was realized in these patients. A Tabotamp^®^ was left in the anal canal and removed the same evening or the day after. All the operations were performed with epidural or general anesthesia (Propofol) plus local anesthesia with Ropivacaine 7.5%.

The patients with associated proctologic pathologies, previous anorectal surgery, diagnosed inflammatory bowel diseases, and chronic assumption of anti-inflammatory therapies were excluded from the study. We obtained specific informed consent from all the patients.

Self-mechanical anal dilatation was indicated only to patients that presented, at the third PO week, a persistent pain, defined as scoring ≥8 on a Visual Analog Score (VAS: 0 min−10 max), a poor quality of defecation, evaluated with Wexner Constipation Score (WCS: 0–30) ≥ 15, and a sphincteric spasm at Rectal Digital Examination (RDE). The edge of eight for VAS to assess chronic pain was decided together with the anesthesiologist and based on a review of the literature.

The dilator has three different sizes (20, 23, and 27 mm in diameter) and has to be soaked in hot water to activate a gel that accumulates and releases heat during dilation, favoring relaxation of the anal sphincter. Before the introduction, the dilatator was lubricated with 2% lidocaine ointment.

All patients were instructed to use the dilator by a member of the surgical team. Self-mechanical anal dilatation had to be performed in Sims' position, introducing the dilatator for at least 3 min a day, preferably before defecation.

It is realized under medical assistance, to verify the complete introduction of the dilator and to increase compliance of the patient toward this potentially embarrassing maneuver. Patients were invited to use the dilator every day, if possible before defecation to minimize its trauma. The scheme indicates the use of a little size dilator, for 1 week, the medium size, for 2 weeks, and the larger size, for the last 2 weeks, for a total application time of 35 days.

Patients were evaluated in the outpatient clinic at 1st PO week (before the beginning of application), 3th PO week (start of application in Group A), 5th PO week (14 days after start of the dilator in Group A), and 8th PO week (end of the application in Group A) by clinical examination, RDE, a VAS pain score, WCS, and overall satisfaction (0–10) at the end of dilator application. Long terms follow-up was performed by phone calls and, in case of doubts, by clinical examination.

All the patients used also stool softeners and analgesic therapy (paracetamol 1 g, maximum 3 for day and, in case of not controlled pain, tramadol hydrochloride 1–2 mg/kg) and were encouraged to practice warm water baths, keeping a daily diary of medication use. They can also apply the same 2% lidocaine ointment, used to lubricate dilator, after defecation.

Self-mechanical anal dilatation patients (Group A) were 1:1 matched by age, gender, and comorbidities, with a historical cohort of patients treated by hemorrhoidectomy, with the same inclusion criteria (a minimum of three piles removed by radiofrequency, score ≥ 8 on a Visual Analog Score at the third PO week, a Wexner Constipation Score ≥ 15, and a spasm at RDE, not other proctologic diseases) without the use of the anal dilators (Group B).

The primary endpoint of this retrospective case-matched study was the VAS pain score, in particular after 2 weeks of dilation-−5th PO week. Secondary endpoints were WCS, use of analgesic therapy after the operation, overall satisfaction of the patient, anal sphincter spasms and scarring, and the incidence of new pathologies, such as stenosis and fissures, requiring further operations.

Overall satisfaction of the patient was evaluated on a specifically designed Likert scale from 0 (not satisfied) to 10 (extremely satisfied). Anal sphincter spasms and scarring were evaluated by RDE, always by the same doctors (CE, LF), not involved in the previous surgical operation and so blinded, describing paradoxical sphincter contractions, tissue scarring consistency, and anal canal diameter.

We also gave the possibility to resume the use of self-mechanical anal dilation in Group A after the end of application analyzed in the study and collected the data during the long-term follow-up.

Data were analyzed using SPSS for Windows, version 21 (SPSS Inc., Chicago, IL, USA).

Means and SDs were used to report continuous data, while numbers and percentages were calculated for all categorical data. Univariate analysis was performed by Student's *t*-test and ANOVA. A *p* ≤ 0.05 was considered statistically significant for all analyses.

## Results

**Figure 1:**
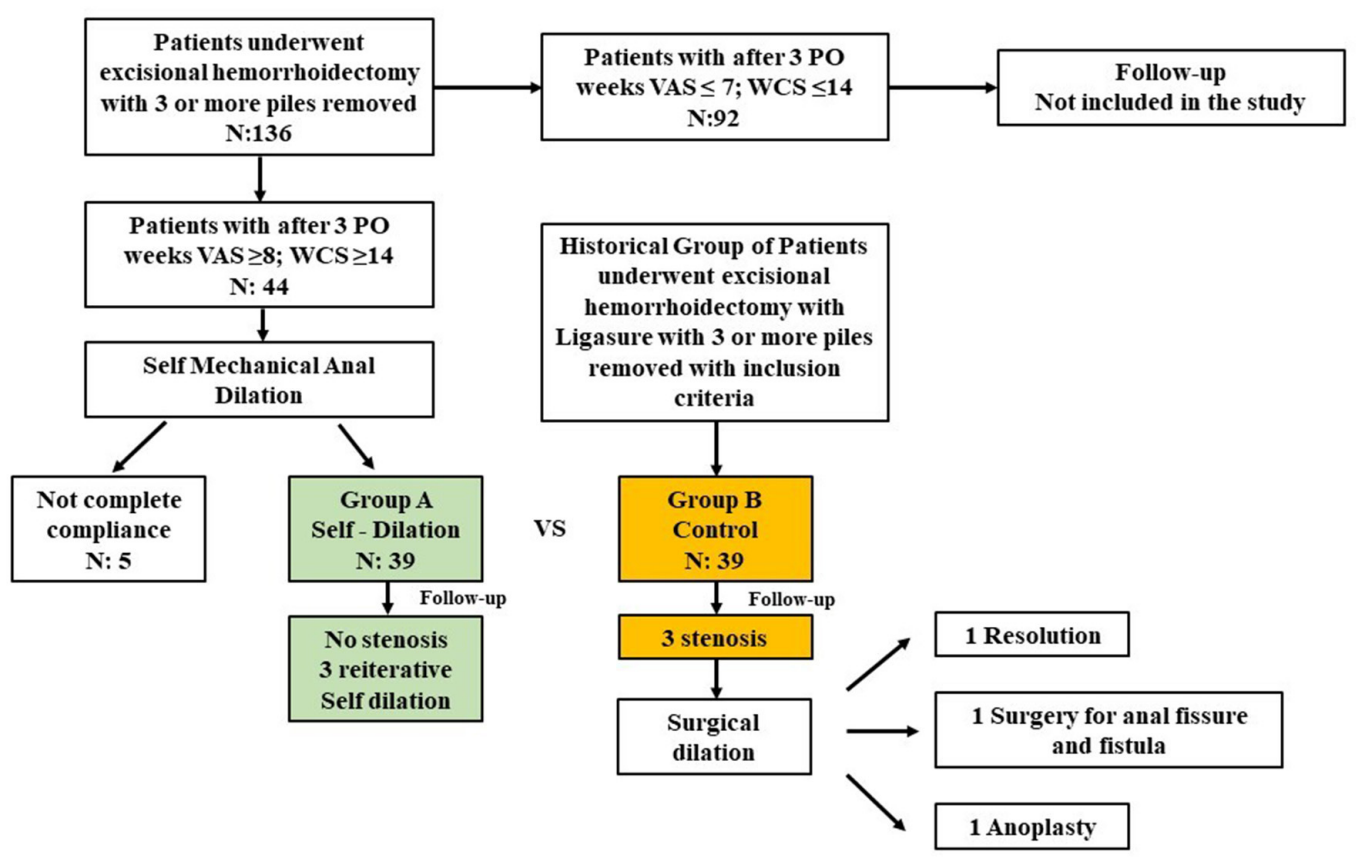
Flow diagram of retrospective matched 1:1 study comparing use or not use of self-dilation after hemorrhoidectomy.

From January 2018 to December 2019, 136 patients underwent radiofrequency hemorrhoidectomy with a minimum of three piles removed at our specialized colorectal surgery unit. Of these, 44 patients met all inclusion criteria (Figure 1) and were indicated for self-mechanical anal dilatation (Group A). Five were excluded during the study because they were not compliant. The final Group A was 39 patients. They were matched with 39 control patients (Group B) from our historical cohort, that had the same inclusion criteria. The baseline presentation was comparable, as shown in Table 1.

**Table 1:** Demographic and clinical characteristics at baseline.

	**Group A** **(Dilation)** **39 pts**	**Group B** **(Control)** **39 pts**
Age		
Mean (SD)	46.7 (13.9)	44.12 (14)
Range	(21–75)	(22–73)
M	19	14
F	20	25
Number of piles removed		
Mean (SD)	3.31 (0.47)	3.18 (0.39)
VAS (1-10)		
Mean (SD)	8.49 (0.7)	8.15 (0.4)
Range	8–10	8–10
WCS (1-30)		
Mean (SD)	19.6 (3.4)	18.7 (3.05)
Range	15–28	15–27

In Group A, the pain decreased very quickly with the use of the dilator, with a mean VAS of 3.25 after 14 days of application (5th PO week) (vs. Group B mean VAS of 5; *p* = 0.00002) and a mean VAS of 1.15 at the end of the application (vs. Group B mean VAS of 3.38 at the 8th PO week; *p* = 0.000000000009). Days of use of analgesic therapy were lower in Group A (mean 12.3 days) than in Group B (mean 17.6 days) (*p* = 0.03).

In Group A, we observed an improvement of the symptoms in a mean period of 14 days after the beginning of the use of the dilator, with a good overall satisfaction vs. lower overall satisfaction in Group B (Group A mean 7.4 vs. Group B mean 5.9; *p* = 0.0000007) and a better quality of defecation (Group A mean WCS 2.87 vs. Group B mean WCS 4.18; *p* = 0.0001).

The digital examination after 8th PO weeks revealed a soft and elastic healing in all the patients of Group A and a persistent reduction of the lumen with fibrosis in 3 (7.7%) patients of Group B (*p* = 0.03) (Table 2). There were no complications with the use of a dilator.

**Table 2:** Early results in the two groups after the dilatation.

**Early results**	**Group A (Dilatan)** **39 pz**	**Group B (Control)** **39 pz**	***P*-value**
VAS at day 0			
Mean (SD)	8.49 (0.7)	8.15 (0.4)	
VAS after 14 days			
Mean (SD)	3.25 (1.51)	5 (1.52)	**0.000002**
VAS at the end of application (35 days)			
Mean (SD)	1.15 (0.92)	3.38 (1.31)	**0.000000000009**
Days of use of analgesic therapy from day 0			
Mean (SD)	12.2 (12.1)	17.5 (12.9)	**0.03**
WCS at the end of application			
Mean (SD)	2.87 (1.29)	4.18 (1.49)	**0.0001**
Clinical stenosis at the end of application	0/39	3/39 (7.7%)	**0.03**
Overall satisfaction (8th PO week)			
Mean (SD)	7.4 (1.25)	5.9 (1.09)	**0.0000007**

During the long-term follow-up (mean 14 PO months) we collected other data: in Group A, in case of constipation or narrow stools, self-dilation was resumed together with stool softener for about 15 days. No patient needed a prolongation of analgesic therapy. There was no evidence of stenosis or chronic pain also at the end of the follow-up.

In Group B, during the long-term follow-up, 14 of 39 patients used analgesic therapy for more than 60 PO days; 10 of 39 patients used fiber/laxative supplements for an average of 6 months; 3 of 39 patients (7.7%) presented severe anal stenosis and underwent, at first, anal dilatation for 2 months: one had a satisfying resolution; two patient presented an anal fissure with fistula that needed a surgical operation after 8 months, with fistulectomy, sphincteroplasty, and anoplasty (7); one patient needed a surgical operation with the removal of the scar and anoplasty for the stenosis.

## Discussion

Approximately 10% of all the patients affected by the hemorrhoidal disease were surgically treated (8). Despite the introduction of new procedures (stapled hemorrhoidopexy, trans-anal hemorrhoidal dearterialization), hemorrhoidectomy remains the most common operation performed and the gold standard for recurrence (9). In the case of IV grade, hemorrhoidal disease with three or more piles involving all quadrants, hemorrhoidectomy with radiofrequency allows an easier wide excision with satisfactory hemostasis. This procedure provides the lowest percentages of recurrences but does not eliminate the usual postoperative complications: pain and bleeding. Many attempts are done to minimize PO pain: metronidazole, mesoglycan, or diosmine by mouth and, to reduce the sphincteric spasm, topical use of 2% diltiazem or GTN ointment, botulin toxin injection, and preventive lateral sphincterotomy (10). In a recent trial flavonoid associated with metronidazole seems to reduce pain, and bleeding, after excisional hemorrhoidectomy (11).

After hemorrhoidectomy, another fearsome complication is anal stenosis. It occurs in ~4% of patients, but this percentage rises (12, 13) when a radical hemorrhoidectomy is performed, with three/four piles removed. With the use of radiofrequency tools or harmonic scalpels, the easiness of surgical procedure can paradoxically produce a wide excision of anoderm and rectal mucosa, without adequate “bridges” (14, 15). This can hesitate in anal stenosis; the average time of onset is ~4 weeks. When a conservative approach (stool softeners, diet, analgesic therapy, and dilation) failed, the treatment of anal stenosis can be difficult: a second surgical operation with scar excision, sphincterotomy, y-v anoplasty, or, in some cases, flaps (15, 16) can be performed. Despite the good results and minor complications of these procedures, a second hospital admission is needed, with an extension of healing time and of lost working days. Furthermore, there is an evident lack of consensus about what surgical treatment can be most useful and the success rate depends on different coloproctology units, surgical experience, number of cases treated.

**Figure 2:**
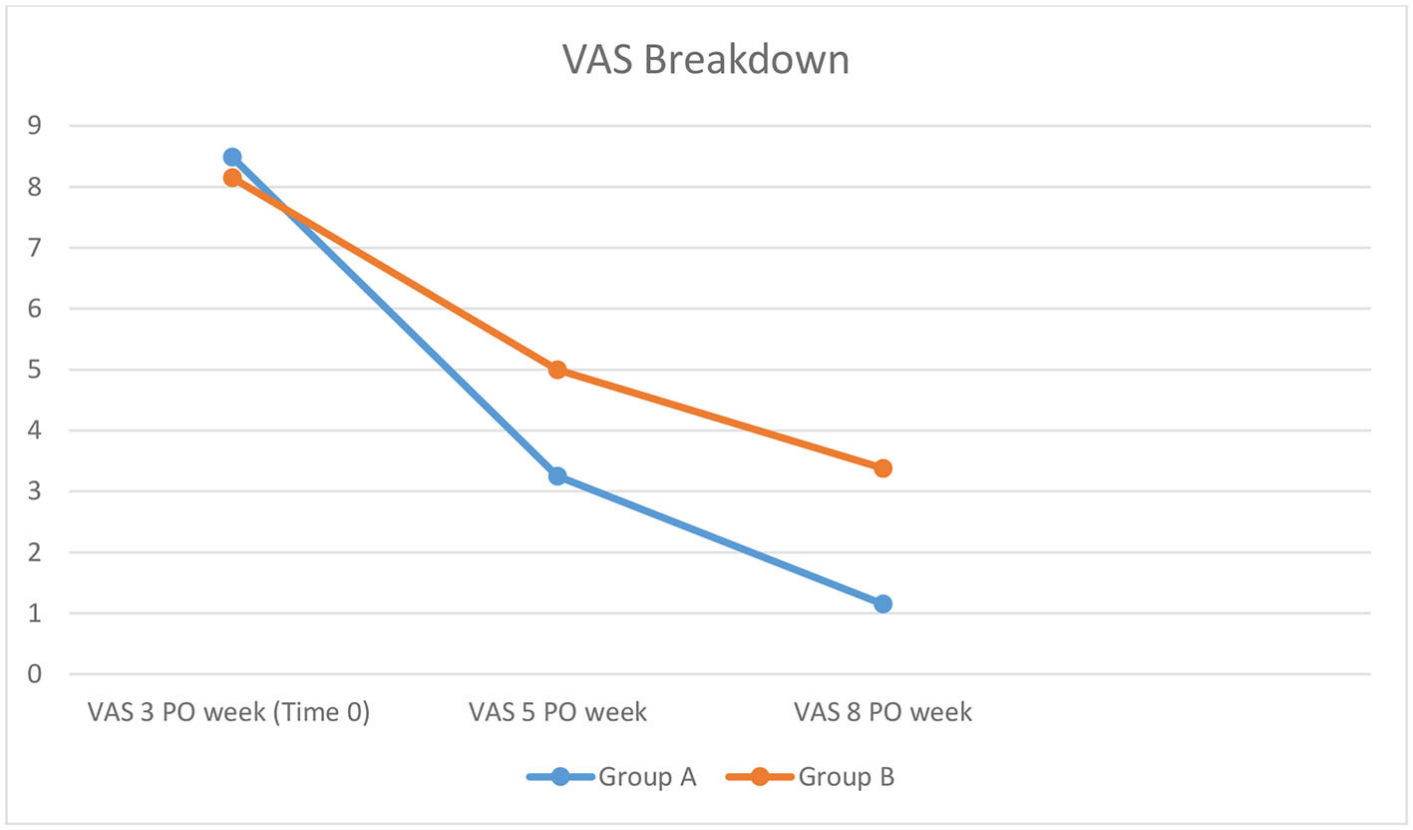
Pain breakdown in the two Groups after the start of dilatation.

According to this study, the use of self-mechanical anal dilation, in an early period after hemorrhoidectomy, is a good clinical practice, especially when a large amount of tissue is removed during the operation. In Group A, we observed the PO pain decreased very quickly with the use of dilator, in the 14 days of application (Figure 2), with a constant improvement and the full recovery of patients. The daily dilation seems to increase soft healing, reduces the spasm and the pain, and allows good healing without retracting scars and anal stenosis. The possibility for patients to perform the procedure by themselves reduces the number of visits of the outpatients. The price of the three dilators is <€40, with an economic return given by the decrease in the use of analgesic therapy and stool softeners.

According to the statistical analysis, the most significant results of self-dilation use are the reduction of pain, improvement of defecation quality, in Group A, especially in the early period, and the absence of late clinical stenosis. These three factors are strictly linked with the quality of life after the surgical operation. Patients accepted this solution sometimes with initial doubts and hesitations, overcome by immediate, evident relief of pain right after the first days of use.

Post-hemorrhoidectomy use of anal dilators is a simple procedure that can result in immediate benefit for the patient; further studies are necessary to confirm these initial, promising results.

The limit of this study is that is retrospective and the number of patients is small, but there will be a wide application in the proctologic unit and the possibility to design new prospective studies in the future.

Self-mechanical anal dilation can guarantee a better late operative course, minimizing the risk of consolidated anal stenosis. The most significant data, despite the small number of patients included in this program, are the breakdown of PO pain with the use of the dilator, improvement in the quality of defecation, and the decrease in the number of PO stenosis after radical hemorrhoidectomy. Avoiding these complications, with a simple tool that can be used by the patient at home, in total privacy, seems really an encouraging result. We need more prospective studies with a larger number of patients enrolled to evaluate the value of this method to reduce PO pain and the risk of post-hemorrhoidectomy stenosis.

## Data Availability Statement

The raw data supporting the conclusions of this article will be made available by the authors, without undue reservation.

## Ethics Statement

The studies involving human participants were reviewed and approved by Department of Surgical Sciences Committee. The patients/participants provided their written informed consent to participate in this study.

## Author Contributions

CE contributed to acquisition, analysis, interpretation of the data, report drafting, and final approval of the version to be published. PM contributed to report drafting, critical revision for important intellectual content, and final approval. KZ contributed to language revision, final draft revision, interpretation of data, and final approval. LF contributed to acquisition and analysis of data and final approval. GN contributed to interpretation of the data, critical revision for important intellectual content, and final approval. DM contributed to design of the work, report critical revision, and final approval of the version to be published. All authors contributed to the article and approved the submitted version.

## Conflict of Interest

The authors declare that the research was conducted in the absence of any commercial or financial relationships that could be construed as a potential conflict of interest.

## Publisher's Note

All claims expressed in this article are solely those of the authors and do not necessarily represent those of their affiliated organizations, or those of the publisher, the editors and the reviewers. Any product that may be evaluated in this article, or claim that may be made by its manufacturer, is not guaranteed or endorsed by the publisher.

## Abbreviations

PO: Postoperative
VAS: Visual Analog Score
WCS: Wexner Constipation Score
RDE: Rectal digital Examination.
